# Transcutaneous Auricular Vagus Nerve Stimulation to Improve Emotional State

**DOI:** 10.3390/biomedicines12020407

**Published:** 2024-02-09

**Authors:** Ainara Aranberri Ruiz

**Affiliations:** Department of Basic Psychological Process and Development, University of the Basque Country, 20018 San Sebastian, Spain; ainara.aranberri@ehu.eus; Tel.: +34-943018311

**Keywords:** emotion, emotional disorders, neuromodulation, transcutaneous auricular vagus nerve stimulation

## Abstract

Emotional experiences are a part of our lives. The maladaptive functioning of an individual’s emotional field can lead to emotional disturbances of various kinds, such as anxiety and depression. Currently, there is an increasing prevalence of emotional disorders that cause great human suffering and high socioeconomic costs. Emotional processing has a biological basis. The major neuroscientific theories of emotion are based on biological functioning, and all of them take into account the anatomy and function of the tenth cranial nerve: the vagus nerve. The vagus nerve connects the subdiaphragmatic and supradiaphragmatic areas and modulates emotional processing as the basis of interoceptive functioning. Auricular vagus nerve stimulation is a new and innovative neuromodulation technique based on the function of the vagus nerve. Several interventions have shown that this new neurostimulation technique is a very promising resource for treating emotional disorders. In this paper, we summarise three neuroscientific theories of emotion, explain what transcutaneous auricular nerve stimulation is, and present arguments for its use and continued research.

## 1. Introduction

Emotional life is a constitutive human reality. As a biological system, emotions take place within our biology; an adaptive emotional life is an indicator of mental health. According to recent data, between 1990 and 2019, the global number of disability-adjusted life years (DALYS) attributable to mental disorders increased from 80.8 million in 1990 to 125.3 million in 2019 [1]; the economic value associated with this burden is estimated to be in the order of five trillion U.S. dollars [2]. Among mental disorders, depressive and anxiety disorders are among the leading causes of DALYS worldwide (ranked 13th and 24th, respectively). In 2019, 301 million people had an anxiety disorder and 280 million had depression. The continued high prevalence of these disorders, along with bipolar and eating disorders, is of particular concern because they also increase the risk of suicide (ranked 18th among the leading causes of death in the 2019 GBD).

These disorders occur in specific neural circuits involved in cortico-amygdalar connections [3,4,5] and are intricately involved in human emotional experience [6,7,8]. Emotional experience is the result of the dynamics generated as a consequence of emotional reactions and the capacity, ability, and willingness to regulate such reactions and the resulting experiences [9]. Emotional response is one of the dimensions that shape temperament, understanding personality as the result of the interaction between temperament and character, where character is the volitional part of personality and temperament; from an epigenetic perspective, it is the part of personality most closely related to our genetic configuration [10].

Therefore, based on these potentials of emotional reactivity in temperament and the capacity for control found in the character dimension of personality, it would be possible to identify, based on scientific knowledge and current technological resources, how to improve emotional states.

To this end, solid explanatory theories based on the current scientific corpus, such as that proposed by LeDoux [11], the theory of constructed emotion [12], and the polyvagal theory [13,14], will be presented below. Additionally, based on the foundations of these theories, together with the description of the technique, arguments will be made for the use of transcutaneous auricular vagus nerve stimulation (taVNS) to improve emotional state.

## 2. The LeDoux Proposal (2021) [11]

LeDoux [11] considers emotions to be conscious experiences that arise in biologically or psychologically significant situations. Concerning consciousness, and based on Endel Tulving’s theory, three states are distinguished: autonoetic, noetic, and anoetic. Each depends on a different kind of memory: autonoetic consciousness depends on episodic memory, noetic depends on semantic memory, and anoetic depends on procedural memory. For example, if you are confronted with the proximity of a snake, using semantic memory, the stimulus is recognised as a snake and is likely to be conceptualised as a potentially dangerous stimulus and situation. The result is a case of noetic awareness of danger, which includes awareness of the relationship between danger and the experience of fear. With the addition of episodic memory, memories of your ‘I’ are added, and ‘you’ become part of the experience: you conceptualise that the snake can harm you. The experience has become an autonoetic state of reflexive self-consciousness in which you are afraid of what might happen to you. Not all autonoetic states are emotional states; however, all emotional states are autonoetic states.

**Figure 1:**
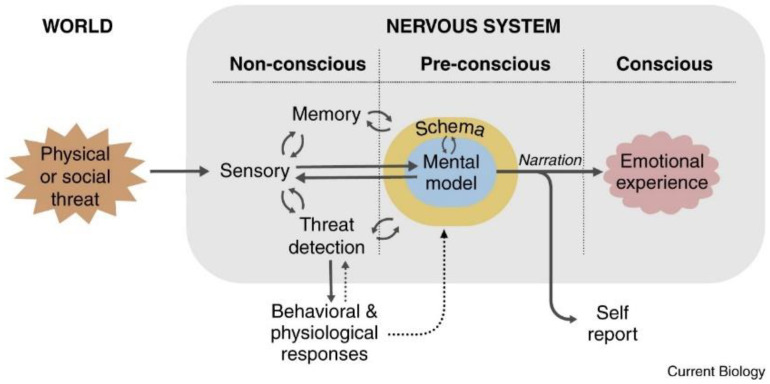
Generation of the emotional experience. Generating emotional experiences. The key to emotional experiences is the integration of perceptual and memory signals with signals related to brain and body states to form situational, self, and emotional schemas. These merge into an unconscious, or more accurately, a preconscious mental model of the emotional situation. The result of the model is a narrative that constitutes the penultimate preconscious antecedent of conscious emotional experience and is also the antecedent of verbal self-report. Adapted from LeDoux [11].

As for the process of emotion generation (see Figure 1), which is a multistage process that develops in three phases of the nervous system (unconscious, preconscious, and conscious), it begins with a stimulus from the world (internal–external) that is meaningful to the subject. Significance is related to risk, which is seen as a reality that threatens the equilibrium of the organism. This significant stimulus is thus processed at an unconscious level by the senses in interaction with memory and the body’s risk detection mechanisms, with the resulting physiological and behavioural responses. The risk detection mechanisms generate automatic physiological and behavioural responses that, together with the processing of memory and information from the senses, are the inputs for the processing that will take place at the preconscious level. Here, with the information from the nonconscious level, schemas of the situation, self, and emotion will emerge; these, in turn, will form a mental model that will be the input received by the conscious level. At the conscious level, the conscious emotional experience is generated through the self-reported narrative of the mental model. The narrative can be about immediate experiences or memories. The conscious emotional experience resulting from the narrative is of a particular categorical type of emotion (e.g., fear, joy, anger, jealousy, envy, pride) or an undefined state of distress or well-being. Because emotional situations can change from one moment to the next, so can schemas, mental models, and narratives. Consequently, emotional experience is dynamic in real time: distress can turn into fear or anger, or fear can turn into anger, jealousy, or shame.

For LeDoux [11,15,16], the key is threat detection, which is a survival mechanism common to all forms of life. In mammals, in particular, a central component of this mechanism is a set of nuclei in the temporal lobe known as the amygdalar complex [17]. The amygdalar complex contains at least 13 separate, distinct nuclei, each with unique patterns of connectivity and functionality [18,19]. These subnuclei are typically grouped into three main divisions: (a) the deep basolateral complex (BLA), consisting of the lateral, basal, and accessory basal nuclei; (b) the superficial cortical nuclei and the nuclei of the lateral olfactory tract; and (c) the centromedial group, which consists of the medial and central nuclei (CeA) [20]. In general, multisensory information from the environment is first received by the amygdala via projections from the nuclei of the thalamus and sensory cortex and transmitted to the BLA and adjacent CeA. The BLA transmits information to cortical regions; this transmission is regulated by excitatory projections from the cortex [21] and associated intercalated cells that can shut down the transmission of nerve impulses between the BLA and CeA [22]. The CeA plays a key role in modulating autonomic and endocrine responses for a range of visceral functions [23]. The cortico-medial region is considered ‘evolutionarily primitive’ due to its connections with the olfactory system. In contrast, the BLA is considered ‘evolutionarily more recent’ due to its predominant coupling with the neocortex, mainly through the medial prefrontal cortex and sensory association areas, as well as other subcortical structures, such as the hippocampus [17]. In this line, Barger et al. [24] conducted a comparative work on the number of neurons in humans and nonhuman primates, the result of which showed a higher cell density in humans concentrated in the lateral nuclei of the amygdala, which have dense connections with different regions of the cortex [17]. It is the different areas in the cortex that are responsible for generating the self-reported narrative of one’s own self-reported emotional experience per se [11]. Concerning emotional experiences, recent studies show that a deficient functional interaction between areas of the prefrontal cortex and amygdaloid nuclei is clearly related to major depressive disorder [5,25], posttraumatic stress disorder (PTSD) [26,27], and various anxiety-related disorders [4,28].

## 3. Theory of Constructed Emotion

The theory of constructed emotion is based on the Bayesian model of brain function. According to this model, as the brain processes sensory inputs, it searches within itself for previous models associated with these incoming inputs, activating those that show the greatest similarity. Based on this information, it makes predictions about what will happen. Thus, when predictions and reality resemble one another, certainty is generated; when they do not resemble one another, uncertainty is generated [7]. While certainty is considered a success, uncertainty is equated with prediction errors (surprises), which the brain uses to revise its models of the world. In all this processing, the brain tries to reduce entropy—prediction errors—by continuously actively adjusting the predictions it makes, i.e., by allostasis. This is because the attempt to reduce surprise is always a consequence of an inherently self-evident mechanism [29]. At the brain level, prediction and prediction error occupy the deep and superficial pyramidal layers of the brain, respectively [30], and from the cortical hierarchy down to the lower levels of the hierarchy, i.e., to the sensory stimuli. At all levels, the goal is to use as few resources as possible to make the connection between sensory input and one’s schema of the world.

In this conception, the theory of constructed emotion [11,12,31] designates as ‘predictive coding’ the process that involves both encoding sensory inputs and the subsequent activation of prior mental models related to the new inputs, and the subsequent process of predicting, with its certainties and surprises. This process also involves the reduction of entropy through allostasis. This predictive coding occurs both concerning the internal environment of the subject’s own body and as a result of the subject’s interaction with the external environment [32,33].

On the one hand, regarding the internal environment, the brain models the world from the perspective of the body’s physiological needs through interoceptive processing [34,35]. In such processing, ascending interoceptive signals carry sensory prediction errors from the internal environment to the brain via lamina I and vagal afferent pathways and are anatomically positioned for modulation by descending visceromotor predictions that control the internal environment [12]. Recent research suggests that interoception is at the core of the brain’s internal model [12,36,37]. Interoceptive sensations are often experienced as low-dimensional affective feelings [12,38]; as such, they possess the properties of affect, i.e., their own valence and arousal [39].

On the other hand, concerning the external world, during the process of socialisation, the subject will integrate, regarding emotional experiences, the properties of the different emotional states granted by the social group to which they belong, integrating within mental schemas the different emotional categories, which will be activated by predictive coding [32]. Specifically, category construction occurs automatically and continuously throughout the lifespan via predictive processing, that is, by predicting, selecting, and processing correctly [12,36,40,41]. Each emotional event begins as a category, constructed as a set of interrelated physical signals that evolve in the brain. The incoming sensory signals, together with the information contained in mental models based on integrated emotional categories, will be the main agents in the allostatic processing aimed at reducing entropy; they will provide the selection of a similar category, whose activation and assembly with the incoming inputs will coordinate motor actions and conscious experience. When unexpected signals arrive from sensory surfaces or expected signals fail to materialise, i.e., when ‘prediction errors’ are made as entropy increases, the brain will attempt to correct its predictions (known as ‘learning’) [32]. Prediction errors are associated with negative valence of emotional experiences, whereas positive valence indicates emotional experiences that resolve uncertainty and provide a sense of control [42]. This links emotional states to uncertainty resolution, neuromodulation, and cortical control of gain [43], giving positive valence to prediction accuracy and negative valence to prediction error.

Based on the interaction between one’s body and the environment, this theory suggests that emotions are created from a dimensional and categorical perspective. The dimensional perspective refers to the dimensions of valence and activation resulting from interoceptive processing. The categorical perspective refers to the process by which the subject, in the face of certain changes in the internal and external world, effortlessly attempts to make sense of the emotional experience based on emotional categories acquired in the process of socialisation [32]. Multiple areas of the brain are involved in the categorical elaboration of emotional experience, and biological circuits are not differentiated for specific emotions [44]. A review by Wager et al. [45] analysed patterns of human brain activity from 148 studies on emotional categories (2159 participants in total). They concluded that emotional categories correspond to predictive patterns in cortical and subcortical areas across multiple brain networks. This means that even category-based emotional experience is rooted in predictive hits and misses, as observed in patterns of brain activity.

## 4. Polyvagal Theory

The polyvagal theory [13,14,46] proposes that the origin of emotional responses resides in automatic and unconscious perceptions made by the autonomic nervous system (ANS) based on perceived safety, risk, or extreme risk. Based on this theory, the ANS is hierarchically structured around the configuration of the vagus nerve. The vagus nerve has multiple innervations and connections to much of the body [47,48]. Structurally, the vagus nerve consists of the ventral and dorsal vagal branches; the dorsal branch lacks myelin and is phylogenetically the oldest, whereas the ventral branch is myelinated and phylogenetically more recent [49,50]. Together with the sympathetic–adrenal axis, which is specific to the sympathetic nervous system, these two complexes form the three neurobiological circuits of the ANS [13].

The ventral-vagal complex (VVC), or myelinated vagus, is activated when the organism perceives safety. It is located in the nucleus ambiguus (NA), and its innervations are directed to supradiaphragmatic areas. From the ventral part of the NA, it exchanges information with the nucleus of the solitary tract (NTS), some cranial nerves, and the sinoatrial node of the heart. With regard to the NTS, it makes connections with the hypothalamus, the limbic system, the periaqueductal grey matter, the amygdala, and various parts of the cortex [47]. In terms of cranial nerves, various innervations of the glossopharyngeal and facial nerves are also found in the NA. Due to the automatic activation of the VVC, the face and voice show prosocial patterns such as smiling and a pleasant tone of voice [13]. Also, concerning the sinoatrial node, at the heart level, the myelinated vagus is an inhibitor of the sympathetic system, acting as a brake, allowing a rapid slowing of the heart rate [50]. The emotional response generated in the organism is typical of a state of uncertainty.

When the body automatically detects a risk, the influence of the ventral vagus disappears, and the sympathetic-adrenal system is activated. The sympathetic-adrenal system is part of the sympathetic nervous system and is considered an adaptive activation system that supports fight–flight behaviour, which is associated with an almost complete withdrawal of the parasympathetic influence of the VVC [13]. The autonomic functions of such a complex are increased heart rate, reduced heart rate variability, the secretion of glucocorticoids and catecholamines, energy production, including glucose, and the conversion of norepinephrine to epinephrine [46]. As the neurobehavioural goal is fight or flight, cognitive function is subordinated to amygdalar function [51]. The emotional response generated is typical of a state of stress.

Finally, when extreme risk is perceived, the dorsal-vagal complex (DVC), or unmyelinated vagus system is activated, mainly innervating subdiaphragmatic areas. The neurobehavioural functions of this complex are immobilisation or passive adaptation, including apparent death and loss of consciousness [46]. Thus, the emotional response generated is related to the features of emotional shock.

Therefore, according to the polyvagal theory, it is assumed that the genesis of emotional experience is found in that automatic perceptual stage and outside the will that the organism performs based on the safety, risk, or extreme risk detected, which generates a primary emotional reaction of well-being, stress, or emotional shock in the organism. The emotional experience is created based on this automatic ANS response.

Having summarised the three theories, the following recapitulation can be made. In the LeDoux model, at the unconscious level of the ANS, the interoceptive state together with risk detection are key; both processes involve the vagus nerve. From the theory of constructed emotion, the affective state of the body (valence and arousal) depends on interoceptive processing and predictive coding, suggesting that the sensory prediction errors of the internal environment are transported by vagal afferents, demonstrating the importance of the vagus nerve. According to polyvagal theory, the vagus nerve is responsible for orchestrating the physiological state of the emotional response based on the vagus nerve’s detection of safety, risk, or extreme risk.

The vagus nerve, the tenth pair of cranial nerves, is the largest component of the parasympathetic nervous system and links the peripheral organs to the brain [52,53], providing information to the brainstem about the state of the sensory organs [54], i.e., the interoceptive state. It also contains efferents originating in the medulla nuclei that regulate cardiac, pulmonary, and gastrointestinal activity [55]. Optimal regulation of this bidirectional centre–periphery pathway (involving interactions between brainstem structures, elements of the central autonomic network, and the prefrontal cortex) is thought to be associated with mental well-being and influence emotional regulatory functions [56].

The question now is, can we improve the emotional state via the vagus nerve? One possible answer seems to lie in an emerging neurostimulation technique, which we will discuss below.

## 5. Transcutaneous Auricular Vagus Nerve Stimulation

**Figure 2:**
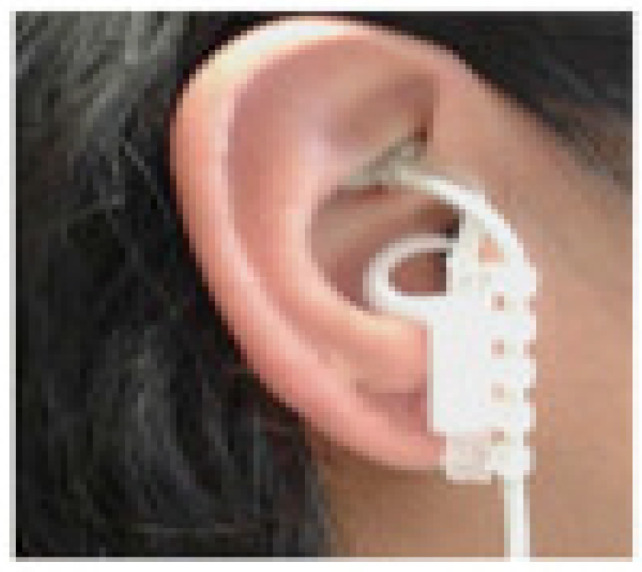
taVNS device. The image shows the electrodes located in the areas innervated by the auricular vagus nerve. Adapted from Warren et al. [59].

**Figure 3:**
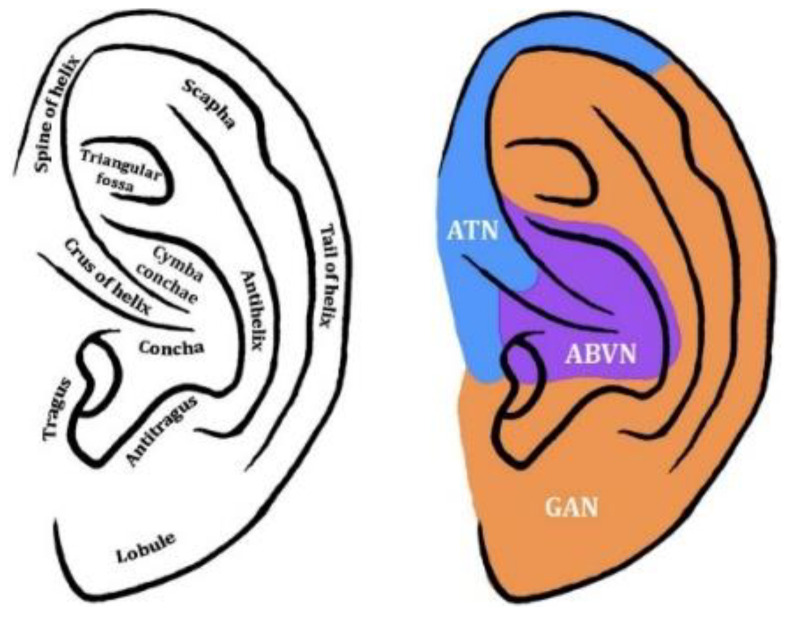
Auricular branch of the vagus nerve. ABVN: auricular branch of vagus nerve. ATN: auricular temporal nerve. GAN: great auricular nerve. The purple areas correspond to those of the auricular vagus nerve. Adapted from Butt et al. [54].

taVNS is a noninvasive brain stimulation technique [57] where electrodes apply electrical impulses to the skin of specific areas of the ear [58,59] (see Figure 2) to stimulate the auricular branch of the vagus nerve, also known as Alderman’s or Arnold’s nerve [60,61]. The location of the auricular vagus nerve was revealed by dissecting the ear of human cadavers [62,63] and mapping the areas of the ear. The outer ear is the only site to which the vagus nerve sends its peripheral branch, the auricular vagus nerve (aVN) (see Figure 3). The aVN is mainly an afferent fibre, innervating the ear and connecting it to the main branch of the vagus nerve [62,63]. Approximately 80% of vagus nerve fibres are considered afferent, while 20% are efferent [54,64]. Like the vagus nerve, the aVN is composed of myelinated A and B fibres and unmyelinated C fibres [58]. Thus, upon application of taVNS, the thick myelinated Ab fibres of Arnold’s nerve are excited, and the afferent signal propagates from the peripheral nerves to the brainstem [65,66,67] and various subdiaphragmatic areas [58,68].

Auricular stimulation is not a new procedure [69]. In one of the earliest Chinese medical treatises written around 2500 years ago—’Huang Di Nei Jing’, The Yellow Emperor’s Classic of Internal Medicine—auricular stimulation using acupuncture is considered a healing practice [70]. Therefore, the proposal for the application of taVNS is novel, nevertheless, its use and development are very old [71]. Until 2000, vagus nerve stimulation could only be performed invasively, via surgical procedures inserting implants into the body that directly stimulate the vagus nerve [55,72]. taVNS was an alternative developed by Ventureyra [73] for epilepsy in 2000, inspired by vagus nerve stimulation (VNS), auricular acupuncture, and the distribution of the aVN in the auricular concha [74]. Several studies concluded that taVNS generally shows similar benefits to internal VNS [69,75]. In addition, unlike internal stimulation, taVNS does not require medical intervention for placement, making it less invasive and less costly [76,77,78]. In addition to auricular stimulation, the vagus nerve can also be stimulated, without the need for surgery, using electrodes attached over the sternocleidomastoid muscle, that is, by cervical vagus nerve stimulation (cVNS). However, the location of the vagus nerve within the carotid sheath under the skin (2 mm), superficial fascia (3–6 mm), and sternocleidomastoid muscle (5–6 mm) [79] can make selective transcutaneous stimulation of the vagus nerve fibres difficult [74], with current products most likely indiscriminately stimulating afferent and efferent fibres alike [53]. Furthermore, in contrast to cVNS, taVNS uses a physiological pathway to activate the NTS and dorsal motor nucleus, which then sends impulses bilaterally to the cardiac surface via the efferent cervical vagus nerves [74]. As a result, this technique avoids the possibility of direct and asymmetric stimulation of cardiac motor efferent fibres, which could lead to adverse cardiac events [80]. For these reasons, taVNS also seems to be an appropriate choice.

As an emerging neuromodulation therapy, taVNS has received particular interest in basic and clinical studies since its introduction [59,81,82] and has been proven to be safe [77,81,82,83]. In a recent meta-analysis and systematic review on the risks of taVNS published by Kim et al. [80], they show that there are no significant differences in terms of risks between the experimental and control groups analysed. Regarding taVNS stimulation parameters, they can vary in current intensity (mA), pulse width (μs), frequency (Hz), duty cycle (s), and session duration (min) [83]. For the mA, μs, and Hz parameters, devices marketed for taVNS are configured with appropriate safety values, although there is still no clear consensus on the optimal parameters [64,84,85].

taVNS stimulation devices are currently CE-marked (but not FDA-approved) for epilepsy, depression, anxiety, pain, and migraine [64]. They are used for epilepsy, major depressive disorder, insomnia, glucose metabolic disorders, pain, stroke, stroke rehabilitation, anxiety, fear, cognitive impairment, cardiovascular disorders, tinnitus, Prader-Willi syndrome, and COVID-19 [69]. The Food and Drug Association (FDA) first approved VNS via a cervical implantable device for the treatment of depression in 1997 [86]. It is currently FDA-approved for the treatment of depression and PTSD [58].

**Figure 4:**
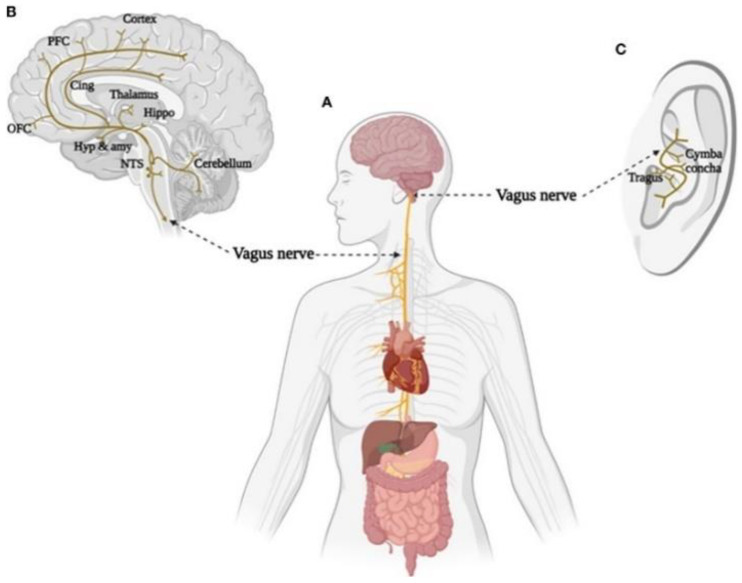
Brain and body projections of the vagus nerve. (**A**) Illustration of the connection between the brain and major body organs via the vagus nerve. (**B**) Brain areas involved in the vagal afferent pathway. Nucleus of the solitary tract (NTS), hypothalamus (Hyp), amygdala (amy), hippocampus (Hippo), cingulate cortex (Cing), orbital frontal cortex (OFC), and prefrontal cortex (PFC). (**C**) Distribution of the vagus nerve in the external ear. Adapted from Zhu et al. [87].

Therefore, the last two decades have seen the rapid development of taVNS devices [55], which, as shown in Figure 4, through electrodes placed on the skin of the ear, over the tragus or cymba conchae, the vagus nerve transmits the impulse received from the taVNS device to stimulate various supradiaphragmatic and subdiaphragmatic areas innervated by the vagus. Specifically, the afferent fibres send the impulse to vagal nuclei connections, to the spinal nucleus of the trigeminal nerve (SNT) in the spinal cord, and to the nucleus of the solitary tract (NST), from which it can modulate activity in widespread subcortical and cortical areas of the brain as components of sensory information are relayed to higher order brain regions (e.g., hippocampus, amygdala, thalamus, and neocortex) [86].

Thus, signals generated in the vagus nerve have the potential to influence a wide range of brain functions [87]. Particularly concerning emotional states, taVNS projects the electrical signal to the NTS and parabrachial nucleus (PBN), from which it projects to cortical and subcortical structures, such as the locus coeruleus (LC), amygdala (A), anterior cingulate gyrus (ACC), and medial prefrontal cortex (mPFC) [83,88,89]. These regions are important nodes of the emotional regulation network, which is associated with the appropriate interpretation of emotions [45,90,91,92,93] and emotional regulation strategies [94,95].

Regarding the projection of the NTS to the LC, it is worth mentioning that the LC is the largest noradrenergic nucleus [96]. Brainstem norepinephrine networks (e.g., LC and NTS) are the main recipients of vagal afferent fibres [53], from which they project to the limbic and hypothalamic regions, where emotion and motivation appear to be controlled [87]. Two key noradrenergic regions that receive these projections are the paraventricular nucleus (PVN) and the supraoptic nucleus (SON), which contain neurons that synthesise the neuropeptide oxytocin and regulate its secretion [97]. taVNS stimulation has been shown to directly modulate the brainstem activity of the LC-norepinephrine network (LC-NE) [77,88,95,98,99]. Indirect noradrenergic physiological markers have been proposed, such as P300, salivary alpha-amylase (SAA), and pupil dilation. Results regarding the effect of taVNS on these physiological markers are inconclusive. On the one hand, laboratory studies show that taVNS affects these noninvasive physiological markers of noradrenergic activity [100,101,102,103]; on the other hand, others show no such effect [97,104,105]. However, the studies showing no effects all use long on/off cycles or continuous stimulation at relatively low stimulation intensities (e.g., 0.5 mA).

As for the subdiaphragmatic projections of the vagus nerve [71], taVNS influences gastrointestinal activity by stimulating the vagus nerve [55,87]; this bidirectional brain-microbiota communication influences cognition, social behaviour, anxiety expression, and stress responses [106]. The vagus nerve plays a key role in such communication [107], influencing central nervous system (CNS) reward neurons [108] based on the aforementioned predictive processing [32]. For example, glutamate released from enteroendocrine cells can activate these vagal afferents [107], as can serotonin [109]. Concerning the gut microbiota, a growing number of studies show that it is associated with anxiety disorders and depression [110,111]. The gut microbiota is a key regulator within the brain–gut axis: bacterial species regulate the production of neurotransmitters and their precursors (e.g., serotonin, GABA, and tryptophan) and can secrete and upregulate essential proteins and metabolites involved in the release of neuropeptides and gut hormones [112,113]. In addition, vagal and spinal afferent pathways mediate neural communication between the gut microbiota and the CNS. Also, the gut microbiota modulates immune signalling from the gut to the brain through cytokine induction [114,115,116]. Simpson et al. [117] conducted a systematic review of the relationship between microbiota and anxiety disorders and depression, concluding that such disorders may be characterised by an increased abundance of proinflammatory species (e.g., Enterobacteriaceae and Desulfovibrionaceae) and decreased production of short-chain fatty acids (e.g., Faecalibacterium). It was also suggested that several taxa and their mechanisms of action may be related to the pathophysiology of anxiety and depression by the transmission of peripheral inflammation to the brain.

## 6. Discussion and Future Research

To summarise the impact of the vagus nerve on the brain–microbiota axis and its relationship with emotional experiences, it is worth mentioning that the first taVNS interventions associated with the emotional domain were aimed at patients resistant to treatment for depression, who, after taVNS, presented improvements in depression-associated symptomatology [117]. The study by Wang et al. [118] found that after one month of taVNS treatment, functional connectivity improved in depressed patients compared with the control/placebo group. Another study on patients diagnosed with depression [119] found that, after two weeks of taVNS, the severity of their depression decreased. This finding was later replicated in a larger sample of patients [120,121].

Regarding anxiety, Lamb et al. [122] conducted a pilot intervention in 22 combat veterans with PTSD, concluding that taVNS affected the systems underlying emotional dysregulation and improved their emotional state. Bottari et al. [123] also conducted a pilot study using taVNS to improve sleep in 13 veterans, with results showing that taVNS can improve sleep depth and stability and increase parasympathetically mediated nocturnal autonomic activity in veterans with PTSD. Schwartz et al. [124] also performed a pilot intervention with taVNS in 10 World Trade Center survivors, who still had PTSD 20 years afterwards, with the intervention reducing their stress responses and hyperarousal. Sanchez-Perez et al. [125] assessed the efficacy of three nerve stimulation modalities in reducing physiological manifestations of stress: taVNS, cervical vagus nerve stimulation (cVNS), and median nerve stimulation (tMNS) in 19 healthy young subjects exposed to three different acute mental and physiological stressors while receiving different stimulations. The results indicated that taVNS and cVNS produced significant changes in the reduction of sympathetic outflow.

The number of both single-session e.g., [126,127,128,129] and multisession e.g., [122,130,131] taVNS interventions is increasing. There is no international consensus on the ideal parameters for taVNS; however, the increasing number of interventions and their positive effects indicate that taVNS is a promising technique for improving emotional states.

Thus, in the future, different taVNS interventions could be developed for specific emotional dysfunction profiles, e.g., transient anxiety states, PTSD, anxiety disorders, etc. In this sense, and considering that taVNS is an emerging technique, there is a need for further research on the effectiveness of the different stimulation parameters used and the duration of treatments and sessions. Possible risks must also be investigated. In this way, the most appropriate type of intervention can be outlined. Additionally, given that the vagus nerve supports the brain–microbiota axis, we also believe that future interventions should analyse both the aforementioned noradrenergic and adrenergic indicators, as well as markers of the human gut microbiota, to obtain further evidence of the effects of taVNS. Finally, given the similar results obtained with the application of cVNS and taVNS [125], it would also be interesting to study cVNS to understand the effects and consequences of the different methods of transcutaneous stimulation.

## 7. Conclusions

Given the complexity of the human emotional experience and its multiple intervening variables, taVNS is presented as a modest neuromodulatory technique for the dimensional physiological improvement of the interoceptive state of a subject’s emotional state. In other words, via taVNS, we can influence and improve the interoceptive state. This more appropriate interoceptive state that, in terms of LeDoux’s model [11], involves a more adaptive reduction of the activation of mechanisms associated with risk detection, i.e., the amygdalar nuclei and adrenal sympathetic axis, and thus, would contribute positively to the construction of a more adaptive narrative of emotional experience. In terms of the theory of constructed emotion [11,12,31], at the dimensional level, this more adequate interoceptive state generates an affective state that is as close as possible to a pleasant valence and an adequate activation of the organism, a consequence of the reduction in surprise and increase in certainty of the organism’s predictive processing. This would allow a possible better execution of the categorical processing of the emotional experience. Finally, from the polyvagal theory [13,46], stimulation of the ventral vagus involves the activation of the vagal brake, which inhibits and prevents the activation of the sympathetic adrenal axis. This generates an interoceptive state of maximum well-being in the organism, which can be achieved in the absence of risk, thus allowing better cognitive processing as a result of the cerebral state of activation of the vagal brake [14].

In summary, together with the improvement of the interoceptive state—a consequence of taVNS—which is fundamental for the adequate management of emotional processing, for subjects to have the most appropriate emotional experiences at any given moment, it will be necessary to develop, in parallel and in the most adaptive way possible, the capacity to generate coherent and healthy narratives; these, from an ethical viewpoint, will allow a nondamaging categorisation. In this way, the scientific knowledge addressed in this work will contribute towards reducing the negative impact of realities generated as a consequence of inadequate management of emotional experiences, both at the individual and social levels.
